# Whole Genome Analysis of Environmental *Pseudomonas mendocina* Strains: Virulence Mechanisms and Phylogeny

**DOI:** 10.3390/genes12010115

**Published:** 2021-01-19

**Authors:** Lidia Ruiz-Roldán, María de Toro, Yolanda Sáenz

**Affiliations:** 1 Área de Microbiología Molecular, Centro de Investigación Biomédica de La Rioja (CIBIR), C/Piqueras 98, 26006 Logroño, Spain; lidiarroldan@gmail.com; 2 Plataforma de Genómica y Bioinformática, Centro de Investigación Biomédica de La Rioja (CIBIR), C/Piqueras 98, 26006 Logroño, Spain

**Keywords:** *Pseudomonas*, microbial ecology, evolution, pan-genome, phylogeny, virulence

## Abstract

*Pseudomonas mendocina* is an environmental bacterium, rarely isolated in clinical specimens, although it has been described as producing endocarditis and sepsis. Little is known about its genome. Whole genome sequencing can be used to learn about the phylogeny, evolution, or pathogenicity of these isolates. Thus, the aim of this study was to analyze the resistome, virulome, and phylogenetic relationship of two *P. mendocina* strains, Ps542 and Ps799, isolated from a healthy *Anas platyrhynchos* fecal sample and a lettuce, respectively. Among all of the small number of *P.* *mendocina* genomes available in the National Center for Biotechnology Information (NCBI) repository, both strains were placed within one of two well-defined phylogenetic clusters. Both *P. mendocina* strains lacked antimicrobial resistance genes, but the Ps799 genome showed a MOB_P3_ family relaxase. Nevertheless, this study revealed that *P. mendocina* possesses an important number of virulence factors, including a leukotoxin, flagella, pili, and the Type 2 and Type 6 Secretion Systems, that could be responsible for their pathogenesis. More phenotypical and in vivo studies are needed to deepen the association with human infections and the potential *P. mendocina* pathogenicity.

## 1. Introduction

*Pseudomonas* is a non-fermenting Gram-negative bacterial genus that colonizes different ecological niches, thanks to their metabolic capacity and broad potential for adaptation to different conditions [1]. This genus includes a wide variety of species, mainly of environmental origin [2]. One of these species is *P. mendocina*, firstly isolated from soil and water samples in the province of Mendoza (Argentina) by Palleroni et al. in 1970 [3]. It is an environmental bacterium, rarely encountered in clinical specimens or reported as a human pathogen, although literature describes some cases of it producing endocarditis or sepsis [4,5].

Unlike the opportunistic pathogen *Pseudomonas aeruginosa*, the pathogenicity mechanisms of *P. mendocina* with respect to human infections have not yet been described. A large number of the species belonging to this genus are marked by their extensive resistance to antimicrobials and the presence of multiple virulence factors [1]. These include adhesins and secretion toxins, effector proteins, proteases, and elastases that facilitate adhesion, modulate host cell signal transduction cascades, and act against the extracellular matrix. Additionally, bacterial pathogens secrete proteins through cell membranes using different secretion systems, acting as toxic products in the host cell cytoplasm [6].

Over the years, next-generation sequencing (NGS) has evolved and incorporated innovations to tackle the complexities of genomes [7]. One of the outcomes of the bacterial post-genomic era was increased interest in the study and comparison of bacterial whole genome sequences in order to define their pan-genomes. A pan-genome is the full set of genes of a given bacterial species or clade [8]. It is constituted by the core genes, i.e., genes found in all strains of the same species, and by accessory genes, i.e., genes present only in some strains. These studies allowed for the characterization of the population structure, evolutionary trajectories, and pathogenicity of different bacterial species of ecological or medical interest, such as the opportunistic pathogen *P. aeruginosa* [9].

Little is known about the genomic characterization of *P. mendocina* species, and this is due to the low number of *P. mendocina* genomes available at the NCBI website at the time that this study was initiated (May 2019). As of December 2019, a total of 19 *P. mendocina* genomes (7 as complete genomes, 3 scaffold, and 9 contigs) were stored in the NCBI database, and all were from an environmental source, whereas 5044 *P. aeruginosa* genomes from different origins (219 complete genomes, 48 chromosome type, 1364 scaffold, and 3413 contigs) were annotated. For this reason, the aim of the present work was to analyze the genome of two *P. mendocina* strains isolated from animal and food samples, and to investigate their phylogenetic relationship with other *P. mendocina* obtained from the NCBI database.

## 2. Materials and Methods

### 2.1. Bacteria Dataset

*P. mendocina* Ps542 and Ps799 strains were isolated from a healthy mallard fecal sample and a lettuce, respectively. Both *P. mendocina* strains were selected from the *Pseudomonas* collection of the Molecular Microbiology group (CIBIR, Spain) by their susceptibility to all tested antipseudomonal agents and their capacity to produce biosurfactant compounds (secondary metabolites).

The mallard (*A. platyrhynchos*) fecal sample was collected in the Castilla-La Mancha region (Spain), and the lettuce (*Lactuca sativa*) from a little shop in the La Rioja region (Spain) in 2016. Both *P. mendocina* strains were identified by classical biochemical methods (Triple Sugar Iron and oxidase reactions), and confirmed by PCR amplification and sequencing of the 16S rRNA fragment [10].

### 2.2. Genome Sequencing, Assembly, and Annotation

Genomic DNA was extracted using the Wizard^®^ Genomic DNA Purification Kit (Promega, Madison, WI, USA), following the manufacturer’s protocol. Quantification of genomic DNA was measured by Qubit fluorimeter (ThermoFisher, Waltham, MA, USA). Genomic libraries were performed with the TruSeq DNA PCR-free Kit (Illumina, San Diego, CA, USA) following the manufacturer’s protocol, and subsequent sequencing was carried out in an Illumina HiSeq 1500 RR 2 × 100 bp (Illumina).

FastQC version 0.11.5 (https://www.bioinformatics.babraham.ac.uk/projects/fastqc/) was used to analyze the quality of raw reads, which were subsequently trimmed and filtered using Trim Galore version 0.4.5 (https://www.bioinformatics.babraham.ac.uk/projects/trim_galore/). Quast (http://quast.sourceforge.net/quast.html) and BUSCO version 4.1.4 (https://gitlab.com/ezlab/busco) [11] analysis were performed in order to investigate the completeness of the sequenced genomes. PLACNETw tool was used to assemble bacterial whole genome and to reconstruct the plasmid content [12]. Genome annotation was subsequently performed using Rapid Prokaryotic Genome Annotation (Prokka version 1.12) [13].

The GenBank assembly numbers of *P. mendocina* available at the time of the study (Dec 2019) and considered as reference genomes in this work are described in Table S1. All of them were *P. mendocina* recovered from non-clinical samples. The reference genomes were annotated using Prokka [13] in order to unify them.

### 2.3. Phylogenetic Analysis

The pan-genome analysis was performed using Roary version 3.11.2 [14]. The phylogenetic tree based on strict core gene alignment was calculated using IQ-TREE [15] (version 1.6.10; bootstraps: 1000).

In order to visually compare the *P. mendocina* genomes, the CGView Comparison Tool [16] was used to map the sequences, using *P. mendocina* ymp as the reference genome, because the complete genome of this strain was the first to be obtained by whole-genome sequencing.

The pairwise average nucleotide identity (ANI) analysis based on BLAST (ANIb), using both *P. mendocina* strains and the nineteen *P. mendocina* reference genomes (Table S1), was conducted using JSpecies version 1.2.1 [17,18] with upper cut-off of more than 95%. The heatmap was performed using iTOL version 4 program [19]. The interspecies ANIb analysis was performed using the twenty-one *P. mendocina* genomes (19 reference genomes, Ps542 and Ps799 genomes) and four *Pseudomonas* spp. reference genomes: *P. aeruginosa* PAO1 (GenBank accession number NC_002516), *P. putida* KT2440 (NC_002947.4), *P. fluorescens* SBW25 (NC_012660), and *P. protegens* Pf-5 (NC_004129.6).

The phylogenetic distances (number of single nucleotide polymorphisms, SNPs) between the *P. mendocina* genomes, from the core genome alignment, were calculated using the ape function of Rstudio (version 3.5.2) [20].

### 2.4. COG Classification, Genomic Islands, and Bacteriophages Prediction

The proteins of each *P. mendocina* genome were classified into clusters of orthologous groups (COGs) functional using the EggNOG mapper web application (version 1.0.3-35-g63c274b) [21].

Genomic islands prediction was analyzed by the IslandViewer 4 [22] web tool, by default settings. DNA blocks of four contiguous open reading frames (ORFs) were predicted as genomic islands [23]. Bacteriophages prediction was realized by PHASTER [24,25] (database last update 20 December 2020, and BLASTp version 2.10.0+) and MetaPhinder [26] (database last update June 2016; minimum thresholds at 70% ID and 70% coverage, and additionally 50% ID and 50% coverage) plus ABRICATE version 0.9.8 (Seeman, T; https://github.com/tseemann/abricate).

### 2.5. Antimicrobial Resistance and Virulence Analysis

The presence of acquired resistance genes was analyzed in the *P. mendocina* genomes by ABRICATE version 1.0.0 (Seeman, T; https://github.com/tseemann/abricate), using the Comprehensive Antibiotic Resistance Database (CARD) version 3.0.8 (27 March 2020) [27]. The prediction of virulence genes was performed using BLAST+ (version 2.10.0+) [28] and a homemade virulence factor database. This database contained many of the virulence genes of *P. mendocina* ymp (GenBank accession number NC_009439) included in the Virulence Factor DataBase (VFDB) (26 April 2020) [29]. Additionally, the poorly annotated genes were curated using the VFAnalyzer software from the Virulence Factor Database (VFDB) [29], comparing the genomes with the *P. mendocina* ymp reference genome (GenBank accession number CP000680.1) and by detecting orthologous genes between *P. mendocina* ymp and *P. aeruginosa* PAO1 (GenBank accession number NC_002516) using OrthoFinder [30].

## 3. Results

### 3.1. Genome Properties

The genome properties of *P. mendocina* Ps542 and Ps799 genomes, as well as the assembly parameters, are described in Table 1. After Illumina sequencing, PLACNETw was used to assemble these genomes. The Ps542 and Ps799 genomes were assembled into 64 and 33 contigs, respectively, including 22 and 15 contigs higher than 1 Kb in size, respectively. The longest contig for each of them measured 1,348,745 and 2,521,352, respectively. The N50 statistics were defined as 467,068 and 734,345 for Ps542 and Ps799, respectively.

Ps542 and Ps799 genomes were constituted by a circular chromosome of 5.2 Mb and 5.4 Mb, and their GC content percentage was 63.04% and 62.63%, respectively. For Ps542, among the 4658 predicted protein coding genes, a total of 3198 (68.6%) were associated with clear functions. For Ps799, 3329 genes from the 4955 predicted coding genes (67.2%) were associated with functions. Therefore, the *P. mendocina* Ps799 genome was larger than the Ps542 genome, including a high number of predicted genes and the presence of a MOB_P_ family relaxase in its chromosome. This site-specific endonuclease belongs to a plasmid backbone and is required for its mobilization. In order to describe this relaxase, an alignment and subsequent tree construction from the first 300 amino acids of several MOB_P_ relaxase models (Table S2), using the LG+I+G4 model, was made with IQ TREE [15,31,32,33]. The relaxase protein belongs to the MOB_P_ family, included in the same cluster as MOB_P3_ relaxases from the reference plasmids, but is placed in its own subcluster (Figure S1). The Ps799 MOB_P_ relaxase showed amino acid changes compared to the remaining MOB_P3_ relaxases, however, the main key residues that define the relaxase motifs were conserved. In addition, a replication initiation protein (RIP) was described also in Ps799. This protein belongs to the Rep3 superfamily protein group, found at 100% identity and 98.55% of coverage in the chromosome of several *Pseudomonas* sp., and one *P. mendocina*, according to a manual BLASTp search. However, despite the presence of this relaxase, no plasmids were detected in Ps542 nor Ps799 genomes.

### 3.2. Phylogenetic Analysis

One of the objectives of this study was the comparison of these two *P. mendocina* genomes with the nineteen reference *P. mendocina* genomes, all of them obtained from the NCBI database (Table S1). The phylogenetic analysis of these *P. mendocina* genomes was carried out by studying their pan-genome. The *P. mendocina* pan-genome of our dataset consisted of 26,392 total genes: 728 belonging to the core genome (99–100% of strains), 362 from the soft core genome (95–98% of strains), and 25,302 genes corresponding to the accessory genome (Table S3).

**Figure 1:**
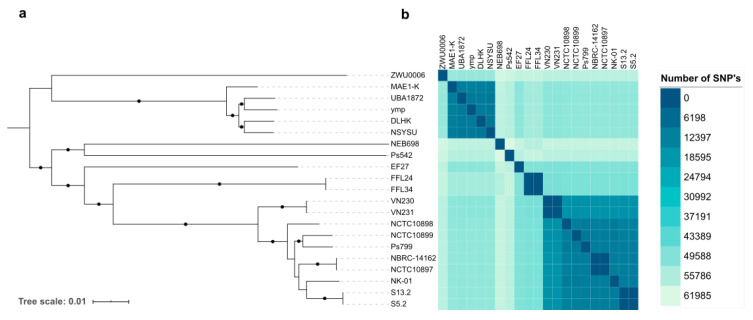
Phylogeny of the two *P. mendocina* genomes (Ps542 and Ps799) and the nineteen *P. mendocina* reference genomes described in the NCBI database (Table S1): (**a**) Core-genome phylogenetic tree based on the essential core genome, using Roary pipeline [14]. Bootstrap values from 90 to 100% were marked as black circles; (**b**) Heatmap of the phylogenetic distances (number of single nucleotide polymorphisms (SNPs)) among the *P. mendocina* core-genomes, using the package ape from Rstudio (version 3.5.2) [20]. The color scale represents the percentage of differences between core-genomes.

**Figure 2:**
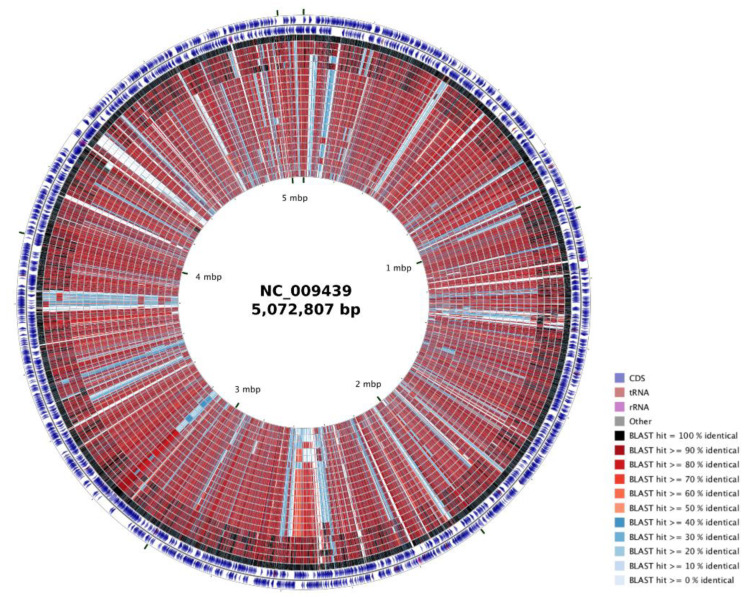
CCT map comparing Ps542 and Ps799 genomes and the nineteen *P. mendocina* reference genomes. The CGView Comparison Tool [16] was used to map the *P. mendocina* sequences, using *P. mendocina* ymp as a reference genome. Prokka-generated GenBank files were used as an input and were converted into CGView XML files for generation of the circular genome comparison map. Starting from the outermost ring, the feature rings depict first the forward strand sequence features, then the reverse strand sequence features. The next 21 rings show regions of sequence similarity detected by BLAST comparisons conducted among CDS translations from all of the compared genomes of *P. mendocina* (ymp, NSYSU, DLHK, MAE1-K, UBA1872, NCTC10899, NK-01, Ps799, NCTC10898, NCTC10897, S13.2, S5.2, NBRC 14162, VN230, VN231, NEB698, FFL24, FFL34, Ps542, ZWU0006, and EF27).

Using their core-genome, from 741,976 nucleotides, a phylogenetic tree was created (Figure 1a). The phylogenetic tree showed the distribution of *P. mendocina* into two clusters, the first one comprised *P. mendocina* ZWU0006, MAE1-K, UBA1872, ymp, DLHK, and NSYSU genomes, and subclassified in two subclusters. The second cluster also comprised two subclusters. The first one only included *P. mendocina* Ps542 and NEB698 genomes, whereas the remaining genomes were included in the second subcluster. Both *P. mendocina* Ps542 and Ps799 genomes belonged to the same cluster. These similarities were also observed in Figure 2, where *P. mendocina* ymp, NSYSU, DLHK, UBA1872, and MAE1-K disclosed high BLAST hits (80–100%) among them.

A clonal relationship was observed between certain *P. mendocina* genomes from the second cluster, e.g., FFL24 and FFL34 (pulp mill wastewater origin, Chile), VN230 and VN231 (pulp mill wastewater, Chile), S5.2 and S13.2 (soil, France), and NBRC-14162 and NCTC10897 (soil, non-determined country) strains. To evaluate the clonality between strains, the phylogenetic distance (number of SNPs) was calculated (Table S4). No SNPs were observed betweenVN230 and VN231 core genomes, only one SNP was found between S5.2 and S13.2 and between NBRC-14162 and NCTC10897, and 5 SNPs were detected between FFL24 and FFL34 genomes (Figure 1b). Analyzing the biological data from the NCBI database, many of the related *P. mendocina* genomes were included in the same BioProject, except for NCTC10897 and NBRC-14162.

In comparison, our *P. mendocina* Ps542 and Ps799 genomes, isolated from a mallard and a lettuce, respectively, showed a high degree of difference between them, based on the high number of different SNPs detected between their core-genomes: 57,468 SNPs (from a total of 741,976 nucleotides).

Additionally, the ANIb values, calculated over the whole genome, were analyzed to determine the genetic identity of all *P. mendocina* genomes. Only a few *P. mendocina* strains showed ANIb values above 95% (Figure S2 and Table S5). Indeed, the differences between the two main clusters were confirmed. *P. mendocina* DLHSK, NSYSU, ymp, MAE1-K, and UBA1872 (first cluster) showed around 97% of ANIb, whereas genomes of the second cluster, *P. mendocina* VN230, VN231, NCTC10898, NCTC10899, NBRC-14162, NCTC10897, NK-01, S13.2, S5.2 (many of them isolated from soil or freshwater sources), and Ps799, showed percentages above 98%. Likewise, *P. mendocina* reference genomes S5.2 and S13.2, and NBRC 14,162 and NCTC10897, displayed ANIb values ranging from 99.9 to 100%, confirming the previously reported results (Figure 1a and Table S5). On the other hand, *P. mendocina* Ps542, NEB698, ZWU0006 EF27, FF124, and FF134 genomes showed identity percentages lower than 85%.

To describe the interspecies identity, an ANIb analysis was performed with four different reference genomes of *Pseudomonas* spp. as outgroups: *P. aeruginosa* PAO1 (GenBank accession number NC_002516), *P. putida* KT2440 (NC_002947.4), *P. fluorescens* SBW25 (NC_012660), and *P. protegens* Pf-5 (NC_004129.6) (Table S6). For these reference genomes, the ANIb values ranged from 74.76% (percentage identity between *P. aeruginosa* PAO1 and *P. fluorescens* SBW25) to 77.82% (percentage identity between *P. aeruginosa* PAO1 and *P. mendocina* MAE1-K). *P. mendocina* genomes showed percentages of identity ranging from 84% to 100% over their whole genome, and SNP values on their core-genome alignment from 0 to 61,985.

### 3.3. COG Classification, Genomic Islands, and Bacteriophages Prediction

**Figure 3:**
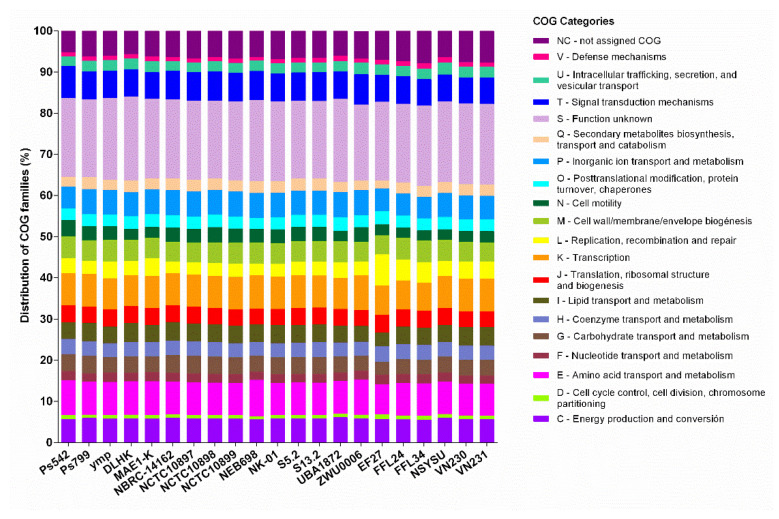
Clusters of orthologous groups (COGs) functional categories and their relative abundances in the *P. mendocina* Ps542 and Ps799 and the nineteen *P. mendocina* reference genomes. Categories with COG < 0.042% (A, B, W, and Z categories) are not represented in the image. All of percentages are described in Table S7.

The protein repertoire of *P. mendocina* Ps542 and Ps799 was compared with the nineteen *P. mendocina* reference genomes. All *P. mendocina* genomes showed similar percentages of COG categories (Figure 3 and Table S7). The main difference was the presence of proteins belonging to “Extracellular structures” (W category) and “Cytoskeleton” (Z category), both detected in *P. mendocina* ZWU0006 reference genome (Table S7). Proteins included in the “Functional unknown” COG category (S category, 18–20%) made up the largest group. The majority of the proteins were distributed among “Amino acid transport and metabolism” (E category, 7–8%), “Transcription” (K category, 7–8%), and “Signal transduction mechanisms” (T category, 6–7%). The remaining categories showed percentages ranging from 1–5% (Table S7). On the other hand, the highest protein abundances in the *P. mendocina* Ps542 genome were observed in M category (5.3%), the “Cell motility” (N category) (4.0%) and T category (7.8%), whereas in the Ps799 genome, they were detected in “Carbohydrate transport and metabolism” (G category) (4.256%) and in “Secondary metabolites biosynthesis, transport, and catabolism” (Q category) (2.925%). No *P. mendocina* genomes showed proteins classified in either “General function prediction only” (R category) or “Nuclear structure” (Y category).

The genomic islands prediction was carried out to analyze part of the accessory genome of the *P. mendocina* genomes. Ps542 and Ps799 exhibited 16 and 18 genomic islands, respectively (Table S8). In the case of Ps542, the regions showed distances between 3871 and 44,758 bp, whereas Ps799 presented regions between 4181 and 49,621 bp. In both cases, non-relevant genes were described in their genomic islands.

On the other hand, no prophages sequences were obtained after using the MetaPhinder program. However, analyzing the data from the PHASTER program, four *P. mendocina* genomes exhibited prophages sequences, including the Ps542 and Ps799 strains (Table S9). Both genomes showed a transposase protein belonging to the *Pseudomonas* phage MD8 (GenBank accession number NC_031091), displaying a 91% of identity (101 of 130 aa). On the other hand, EF27 and MAE1-K presented prophage regions (Table S9), but only in EF27 were two regions with identity higher than 99% and coverage between 97–100% were described: an IS*6*-like element IS*26* family transposase (*Escherichia* phage RCS47; NC_042128) and a Tn*3*-like element Tn*As1* family transposase (*Salmonella* phage SJ46; NC_031129) (Table S9).

### 3.4. Antimicrobial Resistance and Virulence Analysis

According to previous phenotypic studies performed by our group, *P. mendocina* Ps542 and Ps799 strains were susceptible to all antipseudomonal antibiotics tested (piperacillin-tazobactam, aztreonam, cefepime, ceftazidime, imipenem, meropenem, doripenem, netilmicin, tobramycin, gentamicin, amikacin, ciprofloxacin, and levofloxacin). To confirm the presence or absence of resistance genes, the CARD database was used to analyze their resistome (Table S10). No acquired resistance genes were observed in *P. mendocina* Ps542, whereas Ps799 exhibited a *catB11* chloramphenicol resistance gene with one nucleotide deletion, resulting in a truncated protein. On the other hand, three of the nineteen *P. mendocina* genomes carried antimicrobial resistance genes. The EF27 genome harbored the *bla*_CARB-16_ and *bla*_IMP-16_ carbapenemases-encoding genes, the *qnrVC1* quinolone resistance gene, the *arr-4* rifampin ADP ribosyltransferase gene, the *aph(6)-Id* and *aph(3”)-Ib* aminoglycoside phosphotransferase genes, the *ant(2”)-Ia* adenylyltransferase, the *dfrA6* dihydrofolate reductase gene, and the amino acidic substitution T831I in the GyrA DNA gyrase. The VN230 and VN231 carried the *sul1* sulphonamide resistance gene and the *aadA13* aminoglycoside nucleotidyltransferase gene.

To study the virulome, their genomes were analyzed through a homemade virulence database and the VFDB database. The Ps542 and Ps799 genomes were compared with the *P. mendocina* ymp reference genome, as well as the remaining 18 *P. mendocina* genomes (Table S11). One hundred and sixty-five genes belonging to different virulence mechanisms were analyzed. One of the main groups of genes identified were those involved in flagella synthesis, responsible for swimming motility, which were present in all of the *P. mendocina* genomes, especially in Ps542 and Ps799. The genes responsible for the Type IV-pili biosynthesis and twitching motility were highly variable among the *P. mendocina* genomes. Unlike *P. mendocina* ymp, all genomes lacked the *fimT* and *pilA* genes, which are part of the type 4 fimbria biogenesis. On the other hand, the lack of genes responsible for phenazine biosynthesis (*phzA1* to *phzS*) was consistent with the absence of a blue-green color in all of the *P. mendocina* strains studied (Table S11). Additionally, the genes responsible for the synthesis and regulation of alginate production were present in all *P. mendocina* genomes, except for the *mucE* gene, an activator of alginate biosynthesis. Regarding the pyoverdine biosynthesis genes, neither Ps542 nor Ps799 presented these clusters.

Interestingly, neither the most common phospholipases nor proteases were detected in the *P. mendocina* genomes. However, *P. mendocina* ymp showed an orthologous gene, called Pmen_0721 in The Pseudomonas Genome Database, related to the alkaline metalloprotease *aprA* from *P. aeruginosa* PAO1 (PA1249). This gene was annotated as an hemolysin-type calcium-binding protein. Surprisingly, *P. mendocina* Ps542 and Ps799 genomes exhibited the same orthologous gene, called Ps542_01013 and Ps799_01423, respectively, and in both cases it was annotated as a leukotoxin.

Regarding the type VI secretion system, the Hcp secretion island-1 system (H1-T6SS) was highlighted. Only the *P. mendocina* MAE1-K, DLHK, and NSYSU genomes carried the complete operon encoding this system, including the *vgrG1* gene, which is absent in *P. mendocina* ymp (Table S11). Conversely, the NEB698 and Ps542 were unique in that they lacked all of the H1-T6SS operon. In the case of the Type 2 Secretion System (T2SS), all *P. mendocina* strains except EF27 carried the two clusters belonging to this general secretion pathway (Table S11).

Some of these virulence mechanisms are regulated by a quorum sensing (QS) system in *P. aeruginosa*, and are also dependent on the GacA/GacS two-component master regulatory system. No *P. mendocina* genome possessed the three QS systems, *lasI*/*lasR*, *ahlI*/*ahlR*, and *rhlI*/*rhlR* genes, whereas all of them harbored the *gacA*/*gacS* genes.

## 4. Discussion

The NGS is becoming one of the most widely used applications in whole genome sequencing. Using this technology, researchers can obtain the most comprehensive view of genomic information about many bacterial species in order to describe their phylogeny, evolution or pathogenic mechanisms [7]. Unlike genomic studies conducted on species such as *P. aeruginosa* [9,34,35] or *P. putida* [36,37], among others [38], little is known about the species *P. mendocina*. For this reason, this study was carried out to describe and understand the genomic structure of this species.

The two *P. mendocina* strains, Ps542 and Ps799, were isolated from animal and food samples, and their genomes were sequenced using Illumina technology. Once assembled, it was apparent that their genome sizes were bigger than that of the *P. mendocina* ymp reference strain, but similar to that of other *P. mendocina*. This bacterial species showed the smallest genome (5.19 Mb) compared to other *Pseudomonas* species, such as *P. aeruginosa* (6.61 Mb), *P. putida* (6.06 Mb), *P. fluorescens* (6.39 Mb), and *P. syringae* (6.11 Mb) (NCBI Genome, https://www.ncbi.nlm.nih.gov/genome). Nevertheless, the GC content of the different species remained similar, from 58.8% for *P. syringae* to 66.6% for *P. aeruginosa*; the average GC content for *P. mendocina* was 62.5% (NCBI Genome, https://www.ncbi.nlm.nih.gov/genome). One of the main reasons for the differences in genome size could be that the rest of the *Pseudomonas* species had larger accessory genomes including a great number of antimicrobial resistance genes, mobile genetic elements, and genes encoding virulence-related traits [37,39].

Studying the *P. mendocina* phylogeny, all of their genomes available in the NCBI database corresponded to environmental isolates [40,41,42], mainly originated from the Asian continent in the case of the first cluster; in contrast, the second cluster was isolated from Europe, North America, South America, and Asia, showing a wider geographical dispersion. On the other hand, as outlined in a few research articles, a pair of microorganisms can be classified in the same species if their ANI value is higher than 96% [17,43]. Surprisingly, after analyzing the ANIb values, genomes were joined into two specific groups (ANIb values between 97–99% from each one) and some genomes were excluded from both of them, the hypothesis of having *P. mendocina* subspecies in this study was taken into account. However, it is important to remark that all of the reference genomes used in this study were downloaded from the NCBI repository. It is important to well-annotate and increase the number of genomes of this species, including those from different origins, as clinical samples.

Regarding Ps542 and Ps799 genomes, it is important to highlight the presence of a putative MOB_P3_ family relaxase in the *P. mendocina* Ps799. Relaxase recognizes and covalently binds to the origin of transfer site (oriT) of the donor DNA sequence during the transfer process to a recipient cell [44]. This protein catalyzes the initial and final stages during the conjugation process. Additionally, it is the component of the conjugative machinery that is common in plasmid structures. In this case, the MOB_P_ family comprises a cluster of actively evolving relaxases, including distinguishable clades, most of them isolated from soil or manure isolates [31,45]. The MOB_P3_ relaxase proteins have been found mainly in conjunction with the presence of IncX plasmids [31,46,47], but they have also been recently described as part of integrative conjugative elements (ICEs) or integrative mobilizable elements (IMEs), part of the bacterial genome [48]. These genomic but mobile elements are the most abundant conjugative ones in almost all prokaryotic clades [49].

Normally, IMEs, ICEs, and plasmids are responsible for the spread of antimicrobial resistance genes among bacterial species [50]. Despite the appearance of this relaxase, *P. mendocina* Ps542 and Ps799 were susceptible to all antibiotics tested. Consistent with this, in silico analysis confirmed the absence of antimicrobial resistance genes. However, 3 out of the 19 reference genomes showed antimicrobial resistance genes, EF27, VN230, and VN231. Again, this study confirmed the premise of extensive antimicrobial resistance even in clinical and some environmental *P. mendocina* isolates [51,52,53], with antimicrobial susceptibility more common among the environmental isolates [54,55]. However, although *P. mendocina* infections have to date been easily treated with antibiotics, antibiotic pressure and the spread of mobile genetic elements could reverse this in future [55].

In addition to the antimicrobial resistance mechanisms, bacteria can also produce a wide variety of virulence factors as a strategy to evade host immune defenses or colonize new niches [1]. Our *P. mendocina* genomes exhibited a relevant number of virulence genes implicated in flagella motility, type IV pili, and alginate production. Although motility and biofilm are also common in environmental non-pathogenic bacteria, they have been regarded as pathogenicity mechanisms because they are essential for many biological functions, such as the search for nutrients, survival in unfavorable conditions, sexual reproduction, but also for the spreading of diseases. The motility mechanisms are necessary to enable bacterial spread throughout the respiratory tract when establishing infections [56], as well as for dispersion of the bacteria during the last steps of biofilm formation [57]. Crucial to this process is *fliG*, which expresses a protein that locates to the basal body of the flagella and directs rotation [58], detected in all of the *P. mendocina* strains included. However, the missing genes responsible for the core minor pili subunits for Type IV pili (T4P) could avoid the attachment of these strains to host epithelial cells [58,59,60]. It is the same for the missing *mucE* gene, an activator of alginate biosynthesis, which could explain, joined to other mechanisms, the non-mucoid phenotype observed in the *P. mendocina* strains [61]. Regarding the secretion pathway H1-T6SS, whose complete structure was only present in four genomes, including the Ps799, it can act as an antibacterial weapon, giving the bacteria the capacity to inject different effectors to compete with other bacterial cells for the same ecological niche, even the environmental strains [62]. To appropriately coordinate all the virulence factors mentioned above, the GacA/GacS two-component master regulatory system could be enough to modulate the expression of the putative virulence factors in these strains.

In summary, a deep analysis of *P. mendocina* genomes was performed in this study (virulome, resistome, phylogeny) by comparing the genomes of two strains of animal and food origin, with the *P. mendocina* genomes published in the NCBI database. All *P. mendocina* genomes were classified into two phylogenetic clusters. However, the nomenclature of the reference genomes uploaded in an international repository must be reviewed to confirm the species name in this case. Most of the genomes did not harbor antimicrobial resistance genes, but it should be highlighted that all of them possessed putative virulence factors that could be responsible for the pathogenesis in many infections. More phenotypic and in vivo studies are needed in order to further understand the potential role of *P. mendocina* pathogenicity especially in clinical infections in humans.

## Figures and Tables

**Table 1:** Assembly parameters and general features of the *P. mendocina* Ps542 and Ps799 genomes, using PLACNETw (Velvet Optimiser assembly) [12] and Prokka pipeline [13].

	Ps542 Genome	Ps799 Genome
**Assembly parameters**		
K-mer size (bp)	101	101
Number of contigs	64	33
Contig maximum length (bp)	1,348,745	2,521,352
N50 (bp)	467,068	734,345
Total bp assembly	5,187,128	5,446,182
Contigs >1 Kb	22	15
Average insert size (bp)	514 ± 140	529 ± 160
**Genetic elements**		
Size (bp)	5,178,769	5,440,495
GC content (%)	63.04	62.63
Genes	4725	5022
Protein coding genes	4658	4955
Genes with predicted functions	3198	3329
rRNA genes (5S, 16S, 23S)	1, 1, 1	1, 1, 1
tRNA genes	66	66

## Data Availability

All datasets are available. The whole genome data for *P. mendocina* Ps542 and Ps799 were deposited at GenBank using BioProject number PRJNA525577. The raw sequencing data were deposited at NCBI’s Sequence Read Archive (SRA) under accession numbers SAMN11053884 and SAMN11053888, respectively.

## Supplementary Materials

The following are available online at https://www.mdpi.com/2073-4425/12/1/115/s1. Table S1. Genomic characteristics of the nineteen *P. mendocina* reference genomes and their GenBank assembly numbers. Genes and protein data were obtained after Prokka annotation [13]. Table S2. Description of plasmids used in the phylogenetic tree of MOB_P_ relaxases, including their GenBank ID. Table S3. Phylogenetic matrix about the presence/absence of genes in the *P. mendocina* genomes used in this study. Matrix was constructed with Roary (version 3.11.2) [14]. Table S4. Phylogenetic distances (number of SNPs) between the *P. mendocina* Ps542 and Ps799 and the nineteen *P. mendocina* reference genomes, measured in their core genomes. The package ape from Rstudio (version 3.5.2) was used to analyze them. Table S5. Average nucleotide identity (ANIb) values, using BLAST, of the twenty-one *P. mendocina* genomes. Table S6. Average nucleotide identity based on BLAST (ANIb) values, comparing the *P. mendocina* genomes and four different *Pseudomonas* spp. reference genomes. Table S7. Percentages of clusters of orthologous groups (COGs) functional categories in the twenty-one *P. mendocina* genomes. Table S8. Genomic islands (GIs) prediction of the *P. mendocina* Ps542 and Ps799. Islandviewer 4 [22] was used to predict the genomic islands. Table S9. Prophage sentences prediction of the *P. mendocina* Ps542 and Ps799, including the 19 reference genomes downloaded from the NCBI repository. PHASTER [24,25] was used to predict bacteriophages regions. Percentage of identity (Identity (%)) follow the same reference values as Phaster web: Intact (>90%) and Questionable (70–90%). Identity values lower than 70% (Incomplete) were not added. Intact values are highlighted in green. Those phages that showed >90% ID in the Phaster analysis were subjected to a manual BLASTp analysis, indicated in the table with their query coverage. Table S10. Description of the antimicrobial resistance genes described in some *P. mendocina* genomes, using ABRICATE (version 1.0.0) (Seeman, T; https://github.com/tseemann/abricate) and the CARD database (version 3.0.8) [27]. Table S11. Virulence mechanisms of the twenty-one *P. mendocina* genomes obtained from the VFDB database (http://www.mgc.ac.cn/cgi-bin/VFs/genus.cgi?Genus=Pseudomonas) and a homemade BLAST database from the *P. mendocina* ymp genome. Each row corresponded to a virulence gene, each column corresponded to a different *P. mendocina* genome, where the contig number and the start-end of the gene are described. The symbol in parenthesis indicates the gene direction in the contig: +, forward; -, reverse. The ID number of the genes used in the homemade database are in the “ymp” column. Figure S1. Maximum likelihood phylogenetic tree of MOB_P_ family relaxases, including the relaxase of the *P. mendocina* Ps799 genome, using IQ-TREE (version 1.6.10) [15]. The descriptions of the MOB_P_ relaxases from different plasmids are included in Table S2. Color code: black on yellow = invariant amino acids; black on green = key residues that define the relaxase. The MOB_F11_ relaxase from the pDTG1 plasmid (GenBank accession number NC_004999) was used as an outgroup in order to root the tree. Figure S2. Phylogeny of the two *P. mendocina* genomes (Ps542 and Ps799) and the nineteen *P. mendocina* reference genomes described in the NCBI database (Table S1): A. Core-genome phylogenetic tree based on the essential core genome, using Roary (version 3.11.2) [14]. Bootstrap values from 90% to 100% were marked as black circles. B. Heatmap of the average nucleotide identity based on BLAST (ANIb) for each pairwise comparison. Blue color key represents the percentage of differences between genomes.
