# Supplementary material for: Whole Genome Analysis of Environmental Pseudomonas mendocina Strains: Virulence Mechanisms and Phylogeny
Source: Genes (Basel). 2021 Jan 19;12(1):115. doi: 10.3390/genes12010115 (PMC7832885; doi:10.3390/genes12010115)
Supplement: Supplementary file 1 [file genes-12-00115-s001.zip › Supplementary_material.pdf]

SUPPLEMENTARY MATERIAL

Whole genome analysis of environmental *Pseudomonas mendocina* strains: virulence mechanisms and phylogeny.

Lidia Ruiz-Roldán [1,†], María de Toro [2,*] and Yolanda Sáenz [1,*]

[1] Área de Microbiología Molecular, Centro de Investigación Biomédica de La Rioja (CIBIR), C/ Piqueras 98, 26006, Logroño, Spain. lidiarroldan@gmail.com (L.R.R.); ysaenz@riojasalud.es (Y.S.).

[2] Plataforma de Genómica y Bioinformática, Centro de Investigación Biomédica de La Rioja (CIBIR), C/ Piqueras 98, 26006, Logroño, Spain. mthernando@riojasalud.es.

† Current address: Joint Research Unit "Infection and Public Health" FISABIO-University of Valencia, Institute for Integrative Systems Biology I2SysBio (CSIC-UV), Av. de Catalunya, 21, 46020 Valencia, Spain.

* Correspondence: mthernando@riojasalud.es; Tel.: +34 941 27 88 76; Fax: +34 941 27 88 87 (M.dT.); ysaenz@riojasalud.es; Tel.: +34 941 27 88 68; Fax: +34 941 27 88 87 (Y.S.).

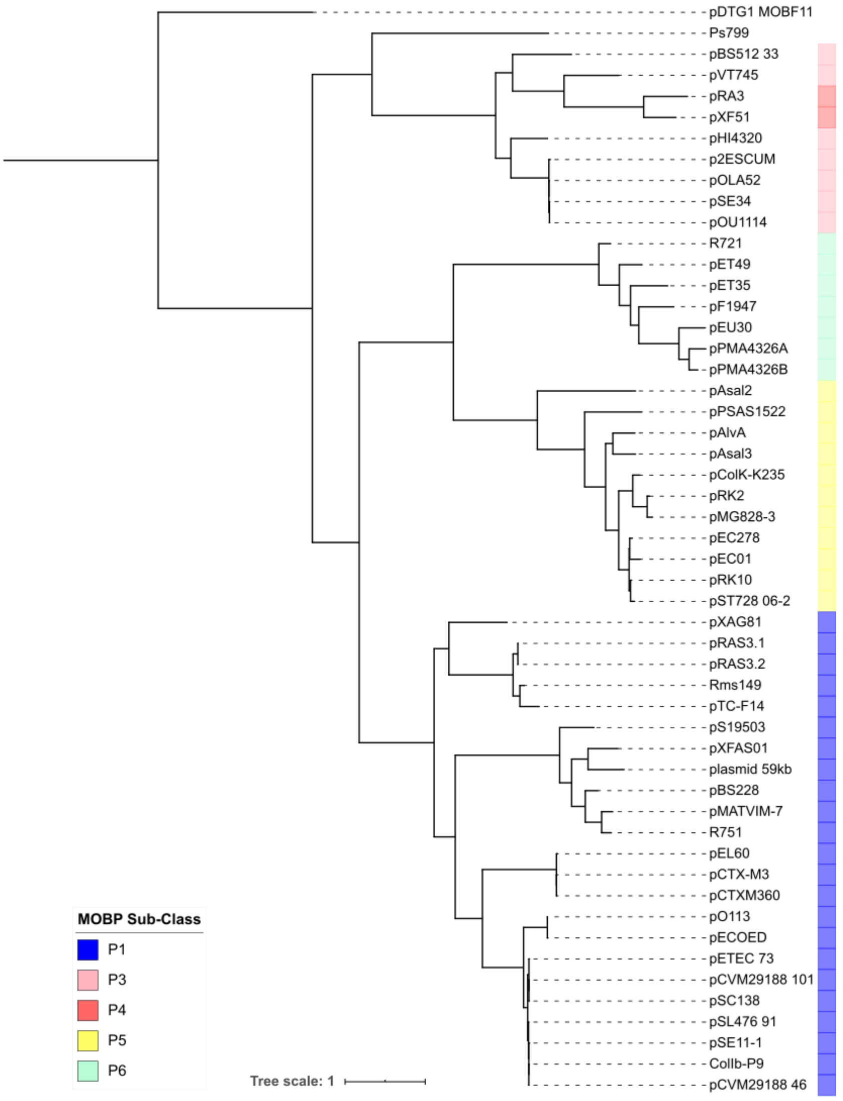

Ps799_MOB$_P$ relaxase amino acidic sequence

MSKGSRPDDELRFRPQPGKPQQRGQPFVNQVLRQANKAGTGKPRKASHQPGASLGRGHVAARFSTKQLPSNARRVTIKTRLVNLRQAGK
RSTLSHLRYIERDGVSREGDPGQAYGPLTDQADLNAFEERGRDDRHQFRFIVSPEDAEQLEDLRTYTRHLMSRMEADLGTRLDWMAVDH
WNTDNPHTHIVLRGKDDTGKDLVIARDYIAEGMRNRASELATEWLGPRTELEIQQSLQREVQQERFTSLDRTLLREHQTGVLSLKSLAN
HPRRQLLIGRLQQLQKLELAYESRPGQWILRDD

Figure S1. Maximum likelihood phylogenetic tree of MOB$_P$ family relaxases, including the relaxase of the *P. mendocina* Ps799 genome, using IQ-TREE (version 1.6.10) [15]. The descriptions of the MOB$_P$ relaxases from different plasmids were included in Table S2. Colour code: black on yellow = invariant amino acids; black on green = key residues that define the relaxase. The MOB$_{F11}$ relaxase from pDTG1 plasmid (GenBank accession number NC_004999) was used as an outgroup in order to root the tree.

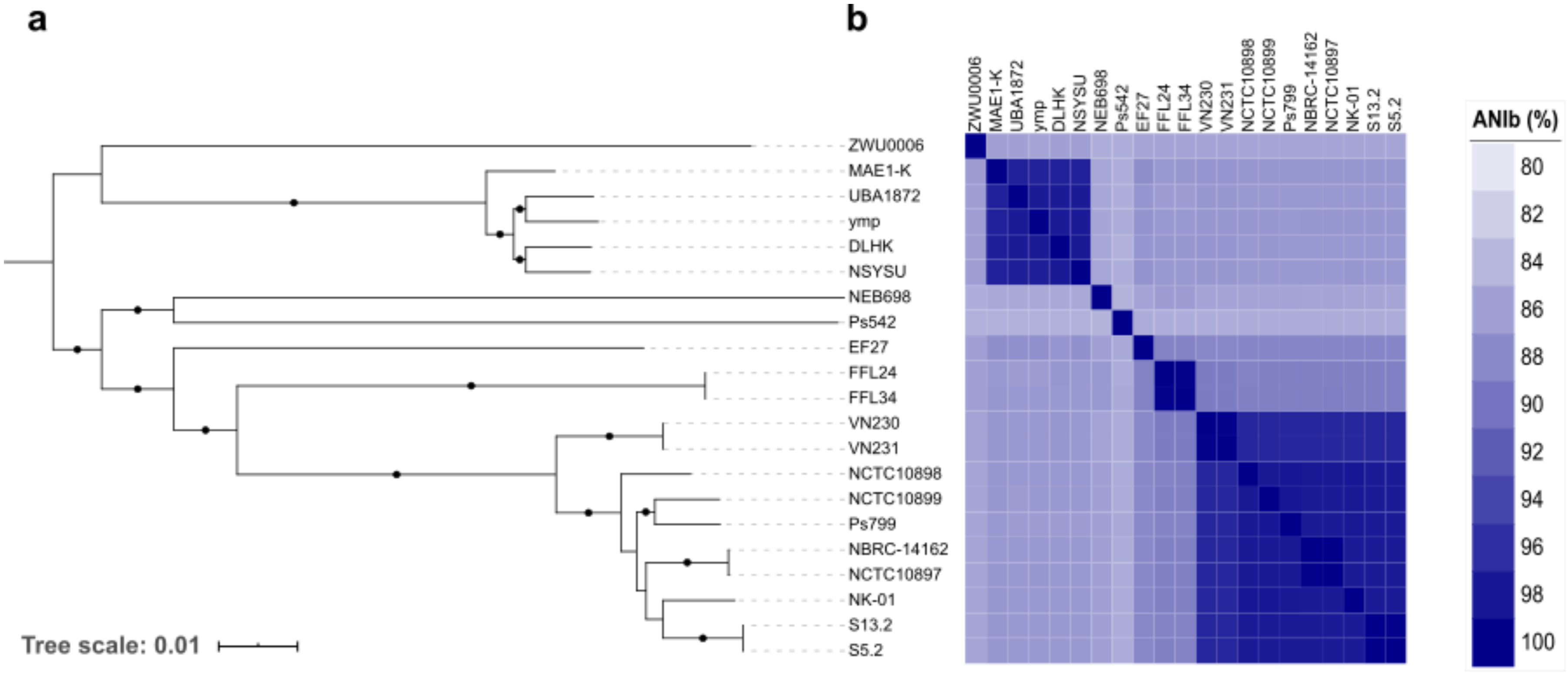

Figure S2. Phylogeny of the two *P. mendocina* genomes (Ps542 and Ps799) and the nineteen *P. mendocina* reference genomes described in the NCBI database (Table S1): **A**. Core-genome phylogenetic tree based on the essential core genome, using Roary (version 3.11.2) [14]. Bootstrap values from 90 to 100% were marked as black circles. **B**. Heatmap of the Average Nucleotide Identity based on BLAST (ANIb) for each pairwise comparison. Blue color key represents the percentage of differences between genomes.
